# Thermal Risk Evaluation of the Fluorobenzotriazolone Nitration Process

**DOI:** 10.3390/molecules30142939

**Published:** 2025-07-11

**Authors:** Yingxia Sheng, Qianjin Xiao, Hui Hu, Tianya Zhang, Guofeng Guan

**Affiliations:** 1 College of Safety Science and Engineering, Nanjing Tech University, Nanjing 211816, China; syx@njtech.edu.cn (Y.S.);; 2 Jiangsu TaiJieZhiBang Testing Technology Co., Ltd., Nantong 226009, China; yyzx@tjzhibang.cn

**Keywords:** fluorobenzotriazolone, nitration reaction, thermal decomposition risk, runaway reaction acceptability, process hazard level

## Abstract

This paper introduces the nitration process of obtaining the synthetic intermediate 1-(2-chloro-4-fluoro-5-nitrobenzene)-4-difluoromethyl-4,5-dihydro-3-methyl-1,2,4-triazol-5(1*H*)-one of pyraclostrobin using raw materials fluorobenzotriazolone, fuming nitric acid, fuming sulfuric acid, and toluene. The exothermic characteristics of the nitration, quenching, extraction, and alkali washing in the nitration reaction were studied, and the thermal decomposition risk of the raw materials and the secondary decomposition risk of the products in the nitration process were evaluated. The results showed that the thermal decomposition risk of the four raw materials was level 1. The acceptable level of runaway reaction in the nitration process was evaluated to be level 2, the acceptable level of runaway reaction in the quenching was level 3, the acceptable level of runaway reaction in the extraction and the alkali washing was level 1, the process hazard level of the nitration reaction and the quenching was evaluated to be level 5, and the process hazard level of the extraction and the alkali washing was level 1. Based on the comprehensive assessment results, targeted risk mitigation and control strategies are proposed to ensure process safety.

## 1. Introduction

Carfentrazone-ethyl is a triazolinone-based herbicide developed by FMC Corporation (USA) and classified as a protoporphyrinogen oxidase (PPO) inhibitor. It holds a significant market share due to its broad-spectrum herbicidal activity [1,2]. There are currently three main synthetic routes for carfentrazone-ethyl [3,4,5]. Carfentrazone-ethyl rapidly inhibits pigment biosynthesis in weeds, leading to growth suppression and eventual plant death [6,7]. It demonstrates effective control of both grassy and certain broadleaf weeds without causing phytotoxicity to crops. Key advantages include high potency, rapid environmental degradation, short half-life, resistance to low temperatures and rainfall, low residue levels, high crop selectivity, minimal toxicity to humans and non-target plants, low environmental persistence, good tank-mix compatibility, and applicability to a wide range of crops [8,9,10,11,12,13,14,15,16].

Thermal hazard evaluation is indispensable for the safe industrial implementation of nitration chemistry. Ray et al. [17] employed differential scanning calorimetry (DSC) and an accelerating rate calorimeter (ARC) to investigate the adiabatic decomposition behavior of 1,2,4-triazol-5-one (TO), the corresponding nitrating liquor, and dicyandiamide (NTO) under strictly adiabatic conditions. By determining key thermal parameters—such as the adiabatic temperature rise (ΔT_ad_) and the maximum temperature of the synthesis reaction (MTSR)—they quantified how feed temperature variations influence the thermal hazard of NTO synthesis, thereby supplying critical safety data for its manufacture, transport, and storage. Complementarily, Yao et al. [18,19] optimized the nitration of m-xylene to nitro-m-xylene via response surface methodology, defining an optimal operating window and calculating a reaction enthalpy of 240.9 kJ mol^−1^ with an adiabatic temperature rise of 98.1 °C. Using a risk matrix in conjunction with the Stoessel criticality diagram, they classified the process thermal risk as acceptable (level 1). Density functional theory calculations further provided activation energies for each elementary step, completing a multiscale risk assessment framework for this nitration route.

In 1988, Gygax introduced MTSR as a quantitative measure of heat accumulation during exothermic processes. He showed that thermal runaway is triggered when the reaction’s heat release rate outstrips the heat removal capacity of the cooling system, i.e., during “cooling failure”. Under adiabatic conditions, the stored reaction enthalpy can drive secondary decomposition reactions, leading to unabated increases in both pressure and temperature until an accident—or even a catastrophe—occurs [20]. Two decades later, Stoessel [21,22] proposed a comprehensive workflow for thermal risk assessment that combines the probability and severity of runaway in a risk matrix format, thereby classifying chemical operations into five hazard levels, from negligible to extreme. This systematic approach now underpins much of the theoretical and practical work on thermal safety evaluation in the chemical process industry. 1-(2-Chloro-4-fluoro-5-nitrophenyl)-4-difluoromethyl-4,5-dihydro-3-methyl-1,2,4-triazol-5(1*H*)-one is a crucial synthetic intermediate in the production of azoxystrobin, obtained via the nitration of fluorobenzotriazolone using mixed acid. To investigate the thermal safety of this nitration process in detail, a series of experiments were designed and carried out, as described in the following section.

## 2. Experimental

### 2.1. Nitration Process of Fluorobenzotriazolone

#### 2.1.1. Process Principle

**Figure 1:**
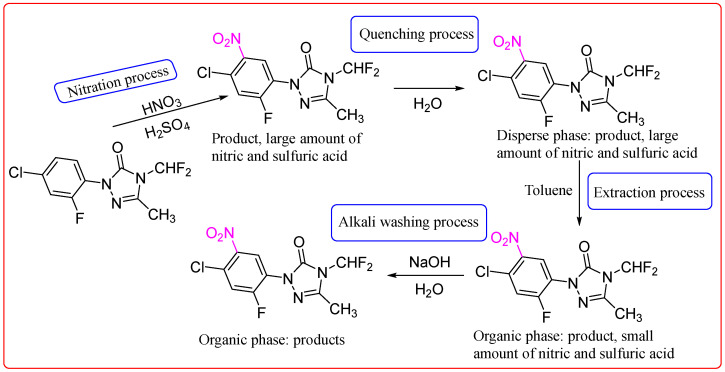
Flowchart of the full process principle of the fluorobenzotriazolone nitration reaction.

The primary raw materials involved in the nitration of fluorobenzotriazolone include fluorobenzotriazolone itself, fuming nitric acid, fuming sulfuric acid, and toluene. The complete process flow for the nitration of fluorobenzotriazolone is illustrated in Figure 1. The nitration reaction proceeds as follows: fuming sulfuric acid serves as the reaction medium into which fluorobenzotriazolone is added and stirred until fully dissolved. Fuming nitric acid is then introduced dropwise under controlled conditions to initiate the nitration. Upon completion, the reaction mixture is discharged into a transit tank and quenched with water to terminate the reaction and reduce thermal risk. The resulting mixture is subsequently extracted with toluene, and the organic phase is subjected to alkaline washing to remove acidic impurities. The final organic layer consists of a toluene solution containing approximately 81% toluene, 18% of the target product—1-(2-chloro-4-fluoro-5-nitrophenyl)-4-difluoromethyl-4,5-dihydro-3-methyl-1,2,4-triazol-5(1*H*)-one—and 1% unidentified impurities.

#### 2.1.2. Process Steps

The nitration process comprises four steps: nitration, quenching, extraction, and alkaline washing. All reaction steps were conducted using a Mettler Toledo reaction calorimeter system (RC1).

(1) Nitration process: The reactor jacket temperature was maintained at 20 °C, and the stirring paddle speed was set to 150 rpm. Initially, 677.10 g of fuming sulfuric acid was charged, followed by the gradual addition of 173.90 g (0.63 mol) fluorobenzotriazolone under continuous stirring until completely dissolved. Subsequently, 40.33 g of 98% nitric acid (0.63 mol) was added dropwise over 45 min at 20 °C. After the completion of the addition, the reaction mixture was held for an additional hour. The maximum system temperature recorded during this step was 27.5 °C.

(2) Quenching process: Water (240.00 g) was added in one portion to the reactor while stirring at 100 rpm using an anchor paddle. The reaction mixture was cooled to 5 °C to complete the nitration step. Following stabilization at 5 °C, 760.0 g of nitration solution was added dropwise over approximately 5 h, during which the system temperature was gradually raised to 30 °C within 4 h. After completing the addition, the reaction was maintained at 30 °C for 1.5 h to ensure system stabilization. Subsequently, key thermodynamic parameters such as specific heat capacity (C_p_) were measured.

(3) Extraction process: A total of 1000.0 g of the quenching solution was charged into the reactor, and the anchor stirring paddle was activated at 150 rpm. The system temperature was maintained at 30 °C. Subsequently, 400.0 g of toluene was added in one portion, and the reaction mixture was heated to 55 °C within 5 min. The reaction was held at this temperature for 1 h to allow stabilization, after which key thermodynamic parameters such as specific heat capacity (C_p_) were measured on the reaction mixture.

(4) Alkaline washing process: A total of 261.80 g of the toluene phase extraction solution was added to the reactor, with stirring maintained at 150 rpm using the anchor paddle. The system temperature was raised to 55 °C and allowed to stabilize. Baseline parameters, including C_p_, were measured on this pre-reaction mixture. Then, 60.0 g of aqueous sodium hydroxide solution was added at once, and the mixture was held at 55 °C for 30 min to complete the reaction. After stabilization, the thermodynamic properties, including C_p_, were again determined for the post-reaction mixture.

To ensure accurate thermal analysis across each stage of the nitration process, various instruments were employed as detailed below.

### 2.2. Experimental Devices

#### 2.2.1. Differential Scanning Calorimetry

To evaluate their thermal stability and potential decomposition hazards, differential scanning calorimetry (DSC) analysis was conducted using a DSC instrument (Mettler Toledo, Greifensee, Switzerland). The results were analyzed using the STARe software package V16.10 to determine the key thermal parameters. The experimental conditions and thermal decomposition data are summarized in Table 1.

#### 2.2.2. Reaction Calorimeter

The exothermic behavior of the fluorobenzotriazolone nitration process was investigated using a reaction calorimeter (RC1) (Mettler Toledo, Greifensee, Switzerland). The test curve included curves for the temperature inside the reaction vessel, jacket temperature, reaction heat release power, total material mass, heat accumulation, heat conversion rate, and specific heat release rate.

#### 2.2.3. Accelerating Rate Calorimetry

The main process streams generated during the fluorobenzotriazolone nitration operation included the nitration product (nitro compound solution), quenching solution, extraction solution, and alkaline wash solution. A secondary decomposition risk assessment was performed using an Adiabatic accelerating rate calorimeter (ARC) manufactured by Thermal Hazard Technology (THT, Milto, Caines, UK). The experimental details are summarized in Table 2.

## 3. Results and Discussion

### 3.1. Thermal Decomposition Risk of Raw Materials

**Figure 2:**
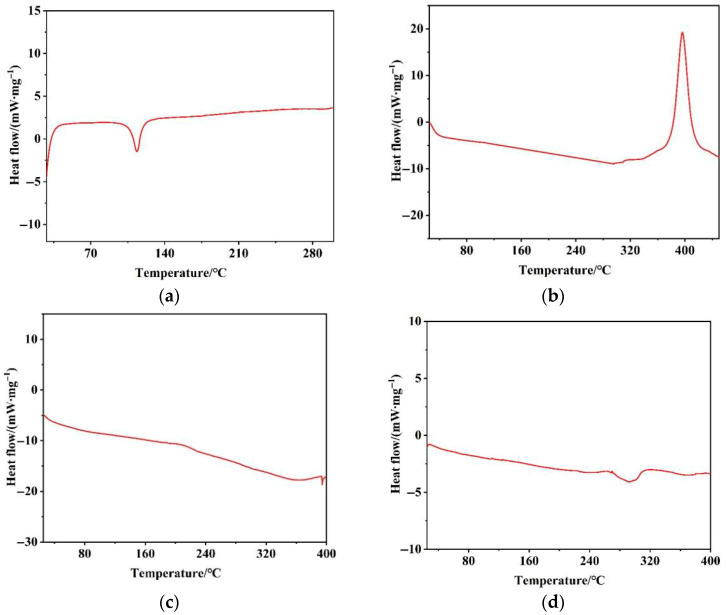
DSC experimental curves of the raw materials: (**a**) fluorobenzotriazolone, (**b**) fuming nitric acid, (**c**) fuming sulfuric acid, and (**d**) toluene.

The DSC test results for fluorobenzotriazolone, fuming nitric acid, fuming sulfuric acid, and toluene are presented in Figure 2.

As shown in Figure 2, no significant exothermic events were observed for fluorobenzotriazolone between 25 and 400 °C. A distinct endothermic peak appeared between 78.94 and 138.74 °C, corresponding to the melting of fluorobenzotriazolone. For fuming nitric acid, an exothermic event was detected within the temperature range of 25 to 400 °C, initiating at 308.17 °C. The exotherm remained incomplete by the end of the test, with an onset temperature (Tonset) of 379.71 °C and a decomposition enthalpy (ΔH) of 488.16 kJ/kg, indicative of thermal decomposition producing nitrogen dioxide, oxygen, and water vapor at elevated temperatures. With further temperature increases, the decomposition of nitric acid continued. Fuming sulfuric acid exhibited a single baseline fluctuation between 367.15 and 387.17 °C, with a Tonset of 368.95 °C and an ΔH of 19.91 kJ/kg. Toluene showed no detectable exothermic signals throughout the 25–400 °C range, demonstrating its thermal stability under the tested conditions. The detailed results are summarized in Table 3. While the thermal decomposition risks of the raw materials were generally low, the reaction processes themselves may pose significant thermal hazards, which are analyzed in the following section.

### 3.2. Thermal Risk of the Nitration Process

**Figure 3:**
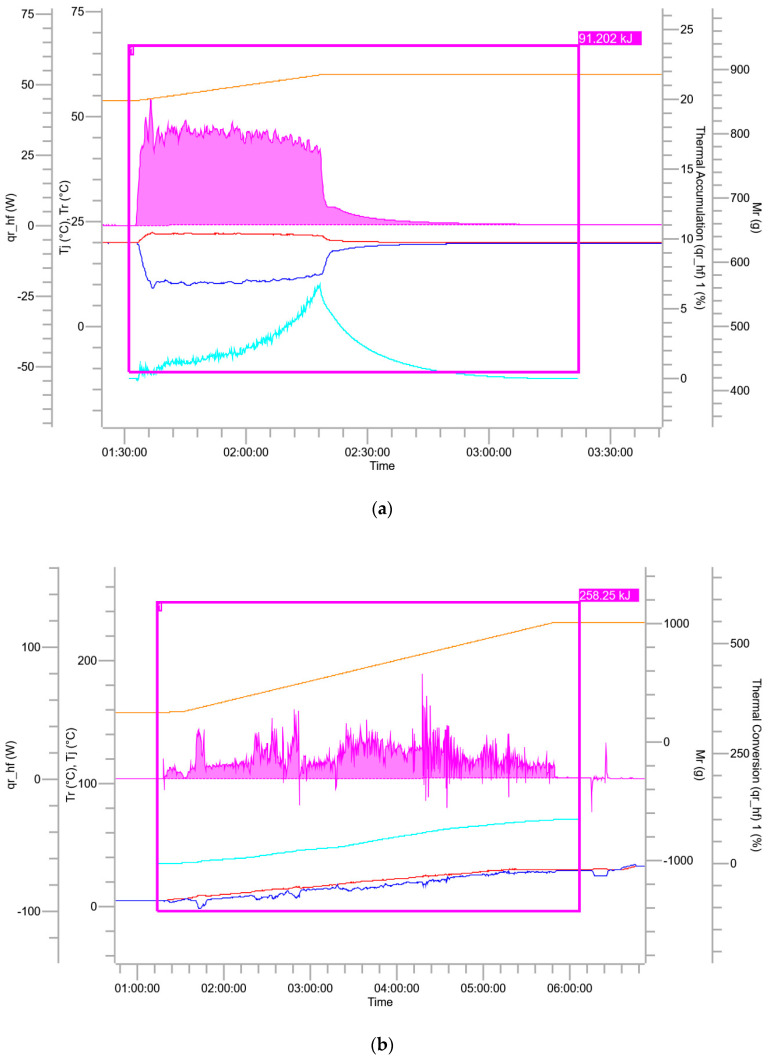

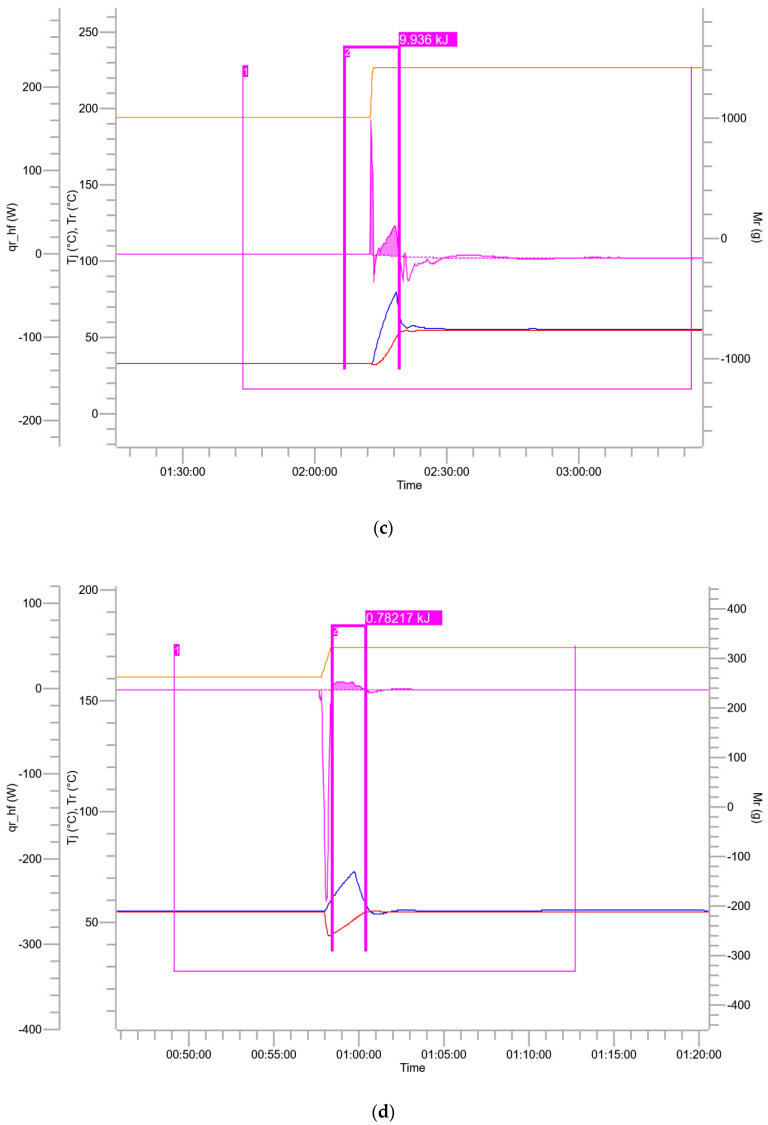
Heat flow profiles obtained from reaction calorimetry experiments: (**a**) nitration step; (**b**) quenching step; (**c**) extraction step; (**d**) alkaline washing step.

The exothermic profiles corresponding to various stages of the reaction process are presented in Figure 3. The calorimetric data include real-time curves for the reactor internal temperature, jacket temperature, reaction heat release rate, total material mass, heat accumulation ratio, thermal conversion rate, and specific heat release rate. The color coding associated with each curve is detailed in Table 4.

As shown in Figure 3, a sharp increase in heat release is observed upon the addition of fuming nitric acid. This phenomenon can be attributed to several factors: fuming sulfuric acid exhibits strong hygroscopicity, and upon contact, fuming nitric acid rapidly absorbs moisture, resulting in a sudden rise in system temperature; under the action of concentrated sulfuric acid, nitric acid undergoes protonation and dehydration, forming nitronium ions (NO_2_^+^), an exothermic process; the nitration reaction itself is highly exothermic, wherein the generated nitronium ions react vigorously with fluorobenzotriazolone, releasing a substantial amount of heat in a short time. Calorimetric analysis of the semi-batch nitration process revealed an adiabatic temperature rise (ΔT_a_d) of 81.86 °C. The subsequent quenching process also exhibited significant thermal hazards, with an adiabatic temperature rise of 191.74 °C. This pronounced exothermicity is primarily due to the mixing of concentrated sulfuric acid and fuming nitric acid with water, which induces rapid dilution and heat release. If the nitration mixture is introduced too quickly or in large volumes, localized overheating may occur, potentially causing violent boiling, splashing, and the generation of corrosive acid mist, posing serious risks to operator safety. However, due to the high thermal conversion rate and low heat accumulation observed in the quenching step, thermal runaway is unlikely if the addition rate is carefully controlled. The extraction and alkaline washing stages were characterized by minimal heat release, with respective adiabatic temperature rises of 4.44 °C and 1.22 °C, indicating that these steps are inherently safe under the tested conditions.

Based on the experimental operating conditions and test results, the key thermal safety parameters for each stage of the fluorobenzotriazolone nitration process were comprehensively evaluated. These include the apparent heat release (Q_a_), specific heat capacity (C_p_), ΔT_ad_, process temperature (T_p_), MTSR, and maximum technically allowable temperature (MTT). The results are summarized in Table 5. All experiments were conducted under atmospheric pressure. For the nitration step, the MTT was defined as the boiling point of fuming sulfuric acid at atmospheric pressure, i.e., 320 °C. For the quenching, extraction, and alkaline washing stages, the MTT was defined as the boiling point of water under atmospheric conditions, i.e., 100 °C, due to the presence of water in these process streams.

Beyond the primary reaction risks, it is also important to evaluate the secondary decomposition behavior of intermediates and products, particularly under adiabatic conditions.

### 3.3. The Risk of Secondary Decomposition of the Products

**Figure 4:**
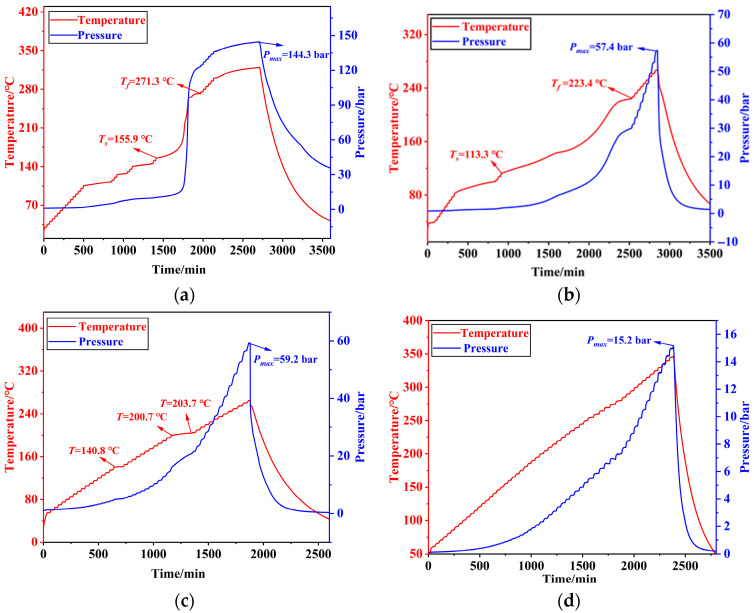
Time–temperature–pressure profiles during adiabatic secondary decomposition of the process solutions: (**a**) nitration; (**b**) quenching; (**c**) extraction; (**d**) alkaline washing.

Figure 4 illustrates the time–temperature–pressure curves under adiabatic conditions for the secondary decomposition of the nitration, quenching, extraction, and alkaline washing solutions. As shown in Figure 4a, five distinct exothermic events were observed in the nitration solution between 30 and 350 °C. The first exothermic event occurred from 106.00 °C to 112.00 °C, releasing 35.05 kJ/kg of heat with an adiabatic temperature rise of 6 °C and an onset heating rate of 0.02 °C/min. The second event spanned from 127 °C to 165.05 °C, with an adiabatic temperature rise of 1 °C and heat release of 5.85 kJ/kg. The third event was detected between 140.9 °C and 143.9 °C, exhibiting an adiabatic temperature rise of 3 °C and heat release of 17.52 kJ/kg. The fourth and most significant exotherm occurred from 155.9 °C to 271.3 °C, with an adiabatic temperature rise of 115.3 °C and a heat release of 672.79 kJ/kg. The fifth exothermic event took place between 300.4 °C and 319.4 °C, corresponding to an adiabatic temperature rise of 19 °C and a heat release of 110.97 kJ/kg.

As shown in Figure 4b, the quenching solution exhibited a single exothermic event within the temperature range of 30–250 °C, starting at 101.22 °C and ending at 223.4 °C. This event released 1123.58 kJ/kg of heat, accompanied by an adiabatic temperature rise of 110.02 °C. The maximum temperature rise rate was 0.63 °C/min, occurring at a peak temperature of 176.35 °C.

As shown in Figure 4c, two exothermic signals were detected in the extraction solution between 55 and 265 °C, although no significant exothermic event occurred. A potential exothermic event was detected at 140.8 °C, but no further reaction was recorded until the instrument resumed detection at 200 °C. At this point, a slow exothermic event was detected again, which ended at 203 °C. Between 200 °C and 203 °C, the adiabatic temperature rise was 3 °C, and the exothermic heat release was 13.58 kJ/kg.

As shown in Figure 4d, no significant exothermic signals were detected in the alkaline wash solution between 55 and 350 °C.

Using data obtained from adiabatic accelerated calorimetry and other tests, the time to maximum reaction rate (TMR_ad_) under adiabatic conditions was estimated based on the reaction kinetic model according to the following equation [23]:

$${TMR}_{ad}=\frac{C_{p}RT_{0}^{2}}{q_{T,0}E}$$

where C_p_, specific heat capacity of the reaction system, kJ/(kg-°C); R, gas constant, 8.314 J/(mol-°C); T_0_, starting process temperature, °C; q_T_,_0_, exothermic rate of the reaction at the temperature of T_0_, W/kg; E, apparent activation energy, kJ/mol.

**Figure 5:**
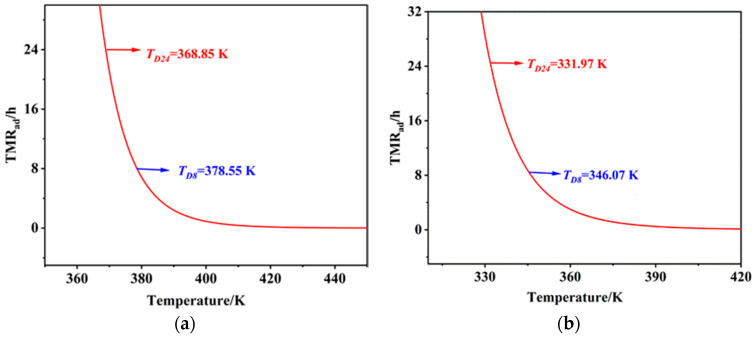
TMR_ad_ versus temperature curves of the reaction products: (**a**) nitration; (**b**) quenching.

According to the TMR_ad_ versus temperature curves obtained from the experimental results, the corresponding temperature at which the time to the maximum rate is 24 h (T_D24_), 8 h (T_D8_), and 1 h (T_D1_) under adiabatic conditions was determined. In cases where no exothermic signal was detected, T_D24_ was conservatively estimated by subtracting 100 °C from the test termination temperature. For the nitration reaction products and the quenching solution, the TMR_ad_ curves and corresponding data are shown in Figure 5. Due to the weak and sparse exothermic signals observed in the extraction solution, T_D24_ was calculated as the test termination temperature minus 100 °C, resulting in a value of 165 °C. No exothermic behavior was detected in the alkaline wash solution throughout the testing range; thus, T_D24_ was similarly estimated at 250 °C based on the termination temperature minus 100 °C.

Following experimental testing, discussion, and analysis, the secondary decomposition heat and the corresponding T_D24_ values of the nitration solution, quenching solution, extraction solution, and alkaline wash solution under adiabatic conditions are summarized in Table 6.

### 3.4. Risk Assessment

#### 3.4.1. Thermal Decomposition Risk

The severity of the thermal decomposition of the raw materials was assessed based on their decomposition enthalpy, while the possibility of decomposition was evaluated according to their onset decomposition temperatures. Subsequently, by integrating both the severity and possibility of decomposition (as shown in Table 7), a thermal decomposition risk matrix was constructed (as shown in Table 8).

Based on DSC testing, the heat released by fuming nitric acid in the temperature range of 25–400 °C was determined to be 488.16 kJ/kg. No significant thermal decomposition was observed for fluorobenzotriazolone, fuming sulfuric acid, or toluene within the tested temperature range. The onset decomposition temperatures of fuming nitric acid and fuming sulfuric acid were identified as 379.71 °C and 368.95 °C, respectively. As shown in Table 7, the decomposition severity of fuming nitric acid was classified as level 2, while fluorobenzotriazolone, fuming sulfuric acid, and toluene were categorized as level 1. The decomposition possibility for all four raw materials was assessed as level 1. Accordingly, the overall thermal decomposition risk levels for fluorobenzotriazolone, fuming nitric acid, fuming sulfuric acid, and toluene were all classified as Risk Class I, as summarized in Table 8.

#### 3.4.2. Runaway Reaction Acceptability

The severity of the runaway reaction is evaluated based on the adiabatic temperature rise of the operation process. The possibility of the runaway reaction is evaluated based on the maximum reaction rate arrival time. Based on the severity and possibility of the runaway reaction (as shown in Table 9), the runaway reaction acceptability evaluation matrix (as shown in Table 10) is constructed.

Based on the thermal safety evaluation of the process, the adiabatic temperature rise (∆T_ad_) values for the nitration, quenching, extraction, and alkaline washing processes were 81.86 K, 197.14 K, 4.44 K, and 1.22 K, respectively. The T_D24_, T_D8_, and T_D1_ for the nitration solution were 95.70 °C, 105.40 °C, and 126.85 °C, respectively, while those for the quenching process were 37.9 °C, 50.51 °C, and 80.1 °C. The T_D24_ values for the extraction and alkaline washing processes were 165 °C and 250 °C, respectively. As summarized in Table 9, the severity of the runaway reactions was classified as level 2 for both the nitration and quenching processes and level 1 for the extraction and alkaline washing processes. The possibility of runaway reactions was assessed as level 3 for the nitration process, level 4 for the quenching process, and level 1 for both the extraction and alkaline washing processes. Consequently, the runaway reaction acceptability classifications, as shown in Table 10, were level 2 for the nitration process, level 3 for the quenching process, and level 1 for the extraction and alkaline washing processes.

#### 3.4.3. Process Hazard Level

According to the thermal safety study results of the nitration process (Table 5), combined with the data from Table 11, the process temperature (T_P_) for the fluorobenzotriazolone nitration reaction is 27.5 °C, with a MTSR of 109.36 °C, a MTT of 320 °C, and a T_D24_ value of 95.7 °C, corresponding to a process hazard level of 5. For the quenching process, the T_P_ is 30.0 °C, MTSR is 227.14 °C, MTT is 100 °C, T_D24_ is 37.9 °C, and the hazard level is also 5. The extraction process exhibits a T_P_ of 55 °C, MTSR of 59.44 °C, MTT of 100 °C, T_D24_ of 165 °C, and a hazard level of 1. Similarly, the alkaline washing process shows a T_P_ of 55 °C, MTSR of 56.22 °C, MTT of 100 °C, and T_D24_ of 250 °C, with a hazard level of 1. These results indicate that the nitration and quenching processes present significantly higher thermal hazards compared to the extraction and alkaline washing processes.

## 4. Conclusions

According to the assessment results, the thermal decomposition risks of the raw materials involved in the nitration process of fluorobenzotriazolone—namely, fluorobenzotriazolone, fuming nitric acid, fuming sulfuric acid, and toluene—are all classified as level 1, indicating that their thermal hazards can be effectively controlled through strict compliance with the relevant regulations, standards, and operational safety protocols during storage and handling.

The acceptable degree of uncontrolled reaction for the nitration process itself is classified as level 2, while that of the quenching process is level 3. Both are evaluated to have a process risk level of 5, suggesting that these steps pose relatively high risks and should be prioritized for process optimization or redesign to mitigate potential hazards. In order to reduce these risks, the process should prioritize controlling the dripping rate of nitric acid in the nitration reaction and the feeding rate of the nitration reaction product in the quenching process, equip the cooling system to promptly remove the heat generated in the reaction process or the quenching process, and set up temperature interlock control. In the nitration process, if the temperature is too high, the nitric acid feed will be cut off, the cooling water will be fully opened, and emergency unloading will be set up. In the quenching process, if the temperature is too high, the feeding of the nitration reaction product will be cut off, the cooling water will be fully opened, and a large amount of chilled water below 10 °C will be injected into the quenching system for cooling. When using a semi-batch reactor, the nitration process of fluorobenzotriazolone is a high-risk process with an adiabatic temperature rise of 86.70 K. It is recommended to use a tubular reactor or a series of continuous reactors to reduce the heat accumulation of the reaction process and improve the intrinsic safety of the nitration reaction. If the semi-batch reactor continues to be used for the nitration reaction, the feeding rate of the nitrating agent must be strictly controlled.

For the extraction and alkali washing processes, the acceptable degree of uncontrolled reaction is rated as level 1, and the overall process risk is evaluated as level 1, indicating relatively low hazard. Nevertheless, these processes should also be equipped with conventional automatic control systems to ensure stable and safe operation through continuous monitoring and control of the process variables.

## Figures and Tables

**Table 1:** Sample information and temperature range for the DSC analysis.

Sample Name	Physical State	Sample Mass (mg)	Test Temperature Range (°C)
Fluorobenzotriazolone	Liquid	3.40	25–400
Nitric acid	Liquid	5.85	25–400

**Table 2:** The parameters for the ARC testing.

Parameters	Fluorobenzotriazolone Nitration Process			
	Nitration	Quenching	Extraction	Alkali Washing
Cuvette material	Hastelloy alloy			
Initial pressure (MPa)	0.10			
Headspace (top space)	Air			
Sample mass (g)	2.31	1.63	2.72	2.09
Specific heat capacity of sample (J·g^−1^·K^−1^)	1.25	1.31	1.60	1.99
Specific heat capacity of cuvette (J·g^−1^·K^−1^)	0.42			
Starting temperature (°C)	30	30	55	55
End temperature (°C)	350	250	265	350
Temperature step increase (°C)	5			
Waiting time at each step (min)	15			
Search time at each step (min)	10			
Temperature rise rate (K·min^−1^)	0.02			

**Table 3:** The test results of the DSC.

Material Name	Exothermic Start Temperature (°C)	Peak Temperature (°C)	Exothermic End Temperature (°C)	Onset of Decomposition Temperature (°C)	Exothermic Heat Release (kJ/kg)
Fluorobenzotriazolone	/	/	/	/	/
Nitric acid	308.17	397.47	-	379.71	488.16

**Table 4:** Color explanation of the heat release curve in the whole reaction process.

Trend	Color	Units
The temperature inside the reaction kettle/Tr	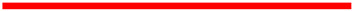	°C
Reaction kettle jacket temperature/Tj	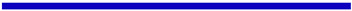	°C
Reaction exothermic power/qr_hf	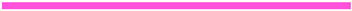	W
The total mass of material in the reaction kettle/Mr	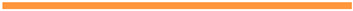	g
Reaction heat accumulation/Thermal Accumulation	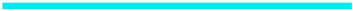	%

**Table 5:** Exothermic characterization parameters and process parameters for the fluorobenzotriazolone nitration process.

Parameter	Nitration	Quenching	Extraction	Alkaline Washing
Q_A_ (kJ)	91.20	258.25	9.94	0.78
C_P_ (kJ·kg^−1^·K^−1^)	1.25	1.31	1.60	1.99
∆T_ad_ (°C)	81.86	197.14	4.44	1.22
T_P_ (°C)	27.50	30	55	55
MTSR (°C)	109.36	227.14	59.44	56.22
MTT (°C)	320	100	100	100

**Table 6:** Secondary decomposition heat and T_D24_ values.

Material Name	Heat of Decomposition (kJ/kg)	*T*_D24_ (°C)
Nitration liquid	825.18	95.70
Quenching liquid	1123.58	37.90
Extraction liquid	13.58	165.00
Alkaline wash solution	-	250.00

**Table 7:** Feedstock decomposition severity and possibility classification [21,22].

**Severity of Raw Material Decomposition**		**Possibility of Raw Material Decomposition**	
**Level**	**Q(J·g^−1^)**	**Level**	**T_onset_ (°C)**
1	Q < 400	1	150 < T_onset_
2	400 ≤ Q ≤ 1200	2	93 < T_onset_ ≤ 150
3	1200 < Q < 3000	3	45 < T_onset_ ≤ 93
4	Q ≥ 3000	4	T_onset_ ≤ 45

**Table 8:** Risk matrix for thermal decomposition of the raw materials.

Risk Level		Severity			
		1	2	3	4
**Possibility**	**1**	I	I	II	II
	**2**	I	II	II	III
	**3**	II	II	III	IV
	**4**	III	IV	IV	IV

**Table 9:** Classification of the severity and possibility for runaway reactions [21,22].

Severity of Runaway Reaction		Possibility of Runaway Reaction	
Level	∆T_ad_	Level	TMR_ad_
1	∆T_ad_ ≤ 50	1	TMR_ad_ ≥ 24
2	50 < ∆T_ad_ < 200	2	8 < TMR_ad_ < 24
3	200 ≤ ∆T_ad_ < 400	3	1 < TMR_ad_ ≤ 8
4	∆T_ad_ ≥ 400	4	TMR_ad_ ≤ 1

**Table 10:** Acceptability for runaway reactions [24].

Risk Level		Severity			
		1	2	3	4
**Possibility**	**4**	II	III	III	III
	**3**	I	II	III	III
	**2**	I	II	II	III
	**1**	I	I	I	II

**Table 11:** Criteria for assessing the process hazards [21,22].

Level	Temperature
1	*T*_p_ ≤ MTSR < MTT < *T*_D24_
2	*T*_p_ ≤ MTSR < *T*_D24_ < MTT
3	*T*_p_ ≤ MTT ≤ MTSR < *T*_D24_
4	*T*_p_ ≤ MTT < *T*_D24_ < MTSR
5	*T*_p_ < *T*_D24_ < MTSR < MTT or *T*_p_ < *T*_D24_ < MTT < MTSR

## Data Availability

The data presented in this study are available on request from the corresponding author.
